# Glycemic, insulinemic and incretin responses after oral trehalose ingestion in healthy subjects

**DOI:** 10.1186/s12937-017-0233-x

**Published:** 2017-02-06

**Authors:** Chiyo Yoshizane, Akiko Mizote, Mika Yamada, Norie Arai, Shigeyuki Arai, Kazuhiko Maruta, Hitoshi Mitsuzumi, Toshio Ariyasu, Shimpei Ushio, Shigeharu Fukuda

**Affiliations:** HAYASHIBARA CO. LTD., 675 Fujisaki, Naka-ku, Okayama 702-8006 Japan

**Keywords:** Trehalose, Insulin, Gastric inhibitory polypeptide

## Abstract

**Background:**

Trehalose is hydrolyzed by a specific intestinal brush-border disaccharidase (trehalase) into two glucose molecules. In animal studies, trehalose has been shown to prevent adipocyte hypertrophy and mitigate insulin resistance in mice fed a high-fat diet. Recently, we found that trehalose improved glucose tolerance in human subjects. However, the underlying metabolic responses after trehalose ingestion in humans are not well understood. Therefore, we examined the glycemic, insulinemic and incretin responses after trehalose ingestion in healthy Japanese volunteers.

**Methods:**

In a crossover study, 20 fasted healthy volunteers consumed 25 g trehalose or glucose in 100 mL water. Blood samples were taken frequently over the following 3 h, and blood glucose, insulin, active gastric inhibitory polypeptide (GIP) and active glucagon-like peptide-1 (GLP-1) levels were measured.

**Results:**

Trehalose ingestion did not evoke rapid increases in blood glucose levels, and had a lower stimulatory potency of insulin and active GIP secretion compared with glucose ingestion. Conversely, active GLP-1 showed higher levels from 45 to 180 min after trehalose ingestion as compared with glucose ingestion. Specifically, active GIP secretion, which induces fat accumulation, was markedly lower after trehalose ingestion.

**Conclusions:**

Our findings indicate that trehalose may be a useful saccharide for good health because of properties that do not stimulate rapid increases in blood glucose and excessive secretion of insulin and GIP promoting fat accumulation.

## Background

Trehalose is a non-reducing disaccharide that consists of two glucose units linked by a α, α-1,1-glucosidic bond. Trehalose is widespread in nature such as plants, fungi and insects, and helps to preserve the life of plants and animals [1, 2]. Although trehalose was previously consumed from natural sources, it has been manufactured from starch through a proprietary enzymatic process since 1995. As a result, trehalose is widely used in a variety of foods to protect against dryness, freezing and osmotic pressure stresses.

In previous studies, we showed that trehalose intake prevented adipocyte hypertrophy and mitigated insulin resistance in mice fed a high-fat diet [3, 4]. In addition, it has been reported that trehalose has various biological effects such as suppression of bone resorption [5] and inflammatory response [6], induction of autophagy [7, 8] and alleviation of Huntington’s disease [9]. Although these effects were found in vitro or in vivo studies, there was little information available about the physiological functions of trehalose in humans.

Oku et al. have reported that trehalose is a carbohydrate that does not raise blood glucose levels quickly and induces lower insulin secretion compared with glucose in female students (age 21.8 ± 5.4) [10]; however, data from a broad age-range of people, including men, remain unknown. Furthermore, the incretin response related to glucose metabolism following trehalose intake has not been examined in human subjects. Incretins are gut hormones that are secreted from enteroendocrine cells into the blood after eating and promote insulin secretion [11]. Gastric inhibitory polypeptide (GIP) and glucagon-like peptide-1 (GLP-1) are main incretins in humans. In recent years, incretin receptor has been detected in many organs, and its various physiological extrapancreatic effects have received a lot of attention [12]. For example, it is known that GIP promotes bone formation [13] and fat accumulation in adipose tissue [14]. This incretin also has been reported to suppress gastric acid secretion in the gastrointestinal tract [15]. In addition, GLP-1 promotes cardioprotection in the heart [16] and delay gastric emptying in the gastrointestinal tract [17]. In this study, we examined glycemic, insulinemic and incretin responses after oral trehalose ingestion in healthy human subjects.

## Methods

### Subjects

Twenty-four healthy Japanese subjects (13 women and 11 men; mean age = 37.8 ± 1.6 years, BMI = 20.7 ± 0.3 kg/m^2^) were recruited. All subjects were informed about the purpose, methods, and possible risks of the study before giving their consent to participate.

This study was approved by the Ethics Committee of Hayashibara Co. Ltd. (approval No. 152) and the Nishi Clinic Ethics Committee (approval No. R1301) (Osaka, Japan). And this study was carried out in Oneness Support Co., Ltd., and the Miura Clinic (Osaka, Japan). All studies were conducted in accordance with the Declaration of Helsinki (adopted in 1964 and revised in 2008) and Japan’s Ethical Guidelines for Epidemiology Research (adopted in 2002 and revised in 2004).

### Test substances

TREHA™ (Hayashibara Co. Ltd., Okayama, Japan) was used as the source of trehalose. It contains more than 98.0% of trehalose dihydrate. Glucose“Yoshida” (Yoshida Pharmaceutical Inc., Tokyo, Japan) consists of 100% glucose anhydrous.

### Study design

Subjects were studied following an overnight fast in the morning on two occasions with an interval of at least 1 week. Trehalose and glucose were ingested during two different trials using a cross-over design.

Although trehalose is hydrolyzed in the small intestine, it was reported that some people caused high osmotic diarrhea because trehalose was not digested completely when large enough amounts were ingested at the same time [10]. In this study, therefore, we decided the quantity of load substance as 25 g, not 50 g generally used for Glycemic Index determination.

Twenty-five grams of trehalose (i.e., 28 g of TREHA™) or glucose were dissolved in 100 mL water and ingested in a gross quantity within 2 min. Venous blood samples were taken before and 15, 30, 45, 60, 90, 120 and 180 min after ingestion. All blood samples were divided at each time point for use in measurements of plasma glucose (Ultraviolet absorption spectrophotometer), serum insulin (Chemiluminescent enzyme immunoassay), plasma active GIP and active GLP-1 (Solid-phase extraction-enzyme-linked immunosorbent assay) by SRL, Inc. (Nara, Japan).

### Data analysis

Time to maximum plasma or serum concentrations (Tmax) and the maximum plasma or serum concentrations (Cmax) were directly obtained from the plasma or serum concentration time-course data.

The area under the curve for blood glucose, insulin, active GIP or active GLP-1 levels was calculated as the incremental area under the response curve (IAUC), ignoring the area beneath the fasting concentration.

### Statistics

Each outcome measure is expressed as the mean ± the standard error (SE). Paired Student’s *t*-tests were applied for the analysis of Tmax, Cmax and IAUC between trehalose and glucose ingestion. For plasma or serum time curves, two–way repeated measures ANOVA (MANOVA) analyze with ingestion, time, and their interaction was used to identify differences between treatments over time. When interaction or ingestion effects were observed, Wilcoxon post hoc analysis was performed to locate these differences. A value of *p* < 0.05 was considered to be statistically significant. All calculations were performed with JMP 9 (SPS Institute Inc.).

## Results

### Analytical subjects of this study

Among 24 participants in this study, one subject was shown to be anemic at blood sampling, two subjects experienced borborygmus and diarrhea after receiving the investigational test substance, and one subject was suspected to have insulin resistance because of high fasting blood insulin levels. Diarrhea symptoms emerged at 50 and 90 min after ingestion of trehalose in the two subjects, with the diarrhea present for several hours. A medical review suggested the presence of osmotic diarrhea due to incompleted digestion in the small intestine and hence the subjects were withdrawn. Therefore, we surveyed the data of 20 subjects (ten women and ten men; mean age = 39.0 ± 1.6 years: range = 23–48, BMI = 20.8 ± 0.3 kg/m^2^: range = 18.3–23.5) as the final analytical results in this study.

### Change of blood glucose and insulin levels

**Fig. 1:**
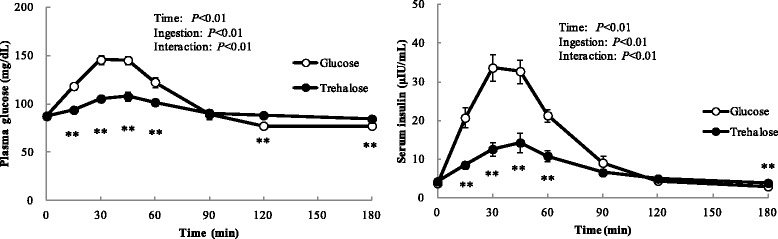
Time-course of blood glucose and insulin levels following ingestion of 25 g of trehalose or glucose. Twenty fasted healthy participants consumed 25 g of trehalose or glucose and changes in concentrations of plasma glucose and serum insulin were measured frequently. Data are expressed as means ± SE (*n* = 20). Data were analyzed with a 2-factor repeated-measures (Ingestion × Tine) ANOVA. ** Significant difference from trehalose and glucose: **p < 0.01 (Wilcoxon post hoc analysis)

Postprandial venous blood glucose and insulin levels are presented in Fig. 1. Trehalose ingestion significantly lowered blood glucose and insulin peaks compared with glucose ingestion. Blood glucose and insulin levels observed in trehalose loading were significantly lower than those in glucose loading at 15, 30, 45 and 60 min after ingestion. Conversely, blood glucose values at 120 and 180 min, and insulin values at 180 min after trehalose ingestion were significantly higher than those after glucose ingestion.

Table 1 showed the metabolic responses after ingestion of trehalose or glucose. Tmax of blood glucose and insulin did not differ between trehalose and glucose loading. Cmax and IAUC(0-2 h) of blood glucose and insulin in trehalose loading were significantly lower than those in glucose loading.

**Table 1:** Indexes of the metabolic response after ingestion of 25 g of trehalose or glucose

	Fasting values	Cmax	Tmax	IAUC (0–2 h)
Blood Glucose	mg/dL	mg/dL	min	mg/dL∙2 h
Glucose	88 ± 1	154 ± 4	39 ± 2	3185 ± 277
Trehalose	87 ± 1	111 ± 4 **	50 ± 6	1201 ± 202 **
Insulin	μIU/mL	μIU/mL	min	μIU/mL∙2 h
Glucose	3.77 ± 0.34	38.1 ± 3.2	36 ± 2	1709 ± 142
Trehalose	4.25 ± 0.50	15.5 ± 2.5 **	39 ± 4	590 ± 101 **
Active GIP	pmol/L	pmol/L	min	pmol/L∙2 h
Glucose	4.2 ± 0.6	49.9 ± 5.8	19 ± 2	2020 ± 212
Trehalose	4.7 ± 1.6	12.6 ± 2.4**	47 ± 10 *	260 ± 50 **
Active GLP-1	pmol/L	pmol/L	min	pmol/L∙2 h
Glucose	1.7 ± 0.1	4.9 ± 0.8	23 ± 2	93 ± 19
Trehalose	2.0 ± 0.2 *	4.6 ± 0.4	32 ± 3 *	116 ± 19

GIP gastric inhibitory polypeptide, GLP-1 glucagon-like peptide-1

Data are expressed as means ± SE (*n* = 20). Differences between glucose and trehalose were assessed using a paired Student’s *t*-test. Significantly different from glucose, *p < 0.05, **p < 0.01

### Change of blood incretin levels

**Fig. 2:**
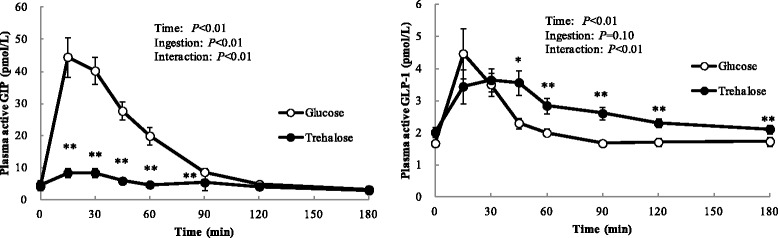
Time-course of blood incretin levels following ingestion of 25 g of trehalose or glucose. Twenty fasted healthy participants consumed 25 g of trehalose or glucose and changes in concentrations of plasma active GIP and plasma active GLP-1 were measured frequently. Data are expressed as means ± SE (*n* = 20). Data were analyzed with a 2-factor repeated-measures (Ingestion × Time) ANOVA. *,** Significant difference from trehalose and glucose: *p < 0.05, **p < 0.01. (Wilcoxon post hoc analysis)

Postprandial venous blood active GIP and GLP-1 levels were presented in Table 1 and Fig. 2.

Cmax of active GIP in trehalose loading was significantly lower than that in glucose loading (Table 1). Furthermore, active GIP levels observed in trehalose loading were lower than those in glucose loading at 15, 30, 45, 60 and 90 min after ingestion (Fig. 2).

Cmax of active GLP-1 was not significantly different between trehalose and glucose loading (Table 1). Moreover, active GLP-1 levels observed at 45, 60, 90, 120 and 180 min after trehalose ingestion were significantly higher than those after glucose ingestion (Fig. 2).

## Discussion

As a result of experiments in healthy human subjects, both blood glucose and insulin levels were found to be significantly lower following trehalose loading compared with glucose loading. Furthermore, active GIP levels were markedly lower following trehalose loading compared with glucose loading. Sex differences were not found in these responses (data not presented).

Diarrhea symptom was observed in the two subjects when 25 g of trehalose were dissolved in 100 mL water and administered in this study. Whereas sixty German subjects were given a 50 g dose of trehalose, on the other hand, diarrhea and malabsorption were not observed in any of the subjects as reported by Bolte et al. [18]. In their study, 400 mL of water was used to administer 50 g of trehalose. In case of Oku et al. [10], furthermore, several dosages of trehalose dissolved in 100–150 mL of tap water were given and no subjects had diarrhea when administered at least 30 g of trehalose. Together with these results, it is possible that diarrhea symptom is caused by the concentration of trehalose solution when given to subject but not total quantity.

Individual blood glucose IAUC after trehalose ingestion was lower than after glucose ingestion. Although a blood glucose IAUC ratio of trehalose loading to glucose loading was individually different, the mean of these values for all subjects was 38% in the present study. This value is equivalent to the glycemic index, which was introduced to classify carbohydrate foods according to the degree of postprandial glycemia. In general, low glycemic index foods improve blood glucose control, the blood lipid profile and fibrinolytic activity in diabetic subjects [19, 20]. Furthermore, postprandial spikes in blood glucose can cause damage to fragile blood vessels in the heart, brain, kidneys, eyes and feet [21–23]. Trehalose might help prevent these risks because it did not rapidly raise blood glucose levels after ingestion, but requires evealuation for potential usefulness as a sweetener for diabetic patients.

Moreover, the mean individual blood insulin IAUC ratio of trehalose loading to glucose loading was 36%. Hyperinsulinemia characterizes impaired glucose tolerance and obesity, and reflects peripheral insulin resistance [24]. Therefore, food materials that do not cause a rapid rise in blood glucose levels and excessive insulin secretion have been expected to prevent onset and progression of lifestyle-related diseases.

Interestingly, active GIP secretion following trehalose loading was conspicuously lower than that of glucose loading. The mean individual blood GIP IAUC ratio of trehalose loading to glucose loading was only 14%. GIP is secreted from K cells, which exist in the upper small intestine, by the stimulation of nutrients such as carbohydrates and lipids [14]. It is thought that ingested trehalose provides little stimulation to K cells during the process of digestion and absorption in the gastrointestinal tract. The lower active GIP concentration found in this study might be one of the mechanisms behind lower insulin release following trehalose ingestion. Recently, a relationship between GIP and obesity has been clarified, and it has been understood that GIP has a physiological role in the nutrient uptake into adipose tissues, thereby linking over nutrition to obesity [14, 25]. Furthermore, it has been reported that GIP receptor-deficient mice fed a high-fat diet were protected from obesity [25]. Restraining GIP signals may lead to the reduction of disease risk such as obesity. In previous reports, we showed that the intake of trehalose suppressed adipocyte hypertrophy and mitigated insulin resistance in mice fed a high-fat diet [3, 4]. Additionally, we confirmed that the consumption of trehalose with a meal three times daily improved glucose tolerance and mitigated the progression of insulin resistance in humans [26]. Low secretion characteristics of GIP may contribute to the suppression of adipocyte hypertrophy and insulin resistance.

In contrast, it is known that GLP-1 protects pancreatic β-cells and suppresses appetite. In this study, although the active GLP-1 levels following trehalose loading were higher than those following glucose loading from 45 to 180 min after ingestion, the levels were very low probably because of rapid disintegration from the enzyme dipeptidyl peptidase-4 (DPP-4). Therefore, we could not argue that trehalose loading led to higher levels of active GLP-1 secretion than glucose loading in this study. A further study is planned in our laboratory.

Recently, DeBosch et al. [8, 27] proposed a model that trehalose inhibits multiple glucose transporter (GLUT) family members through docking in the inward open conformation of glucose transporters. Therefore, trehalose may prevent excessive glucose absorption in the small intestine by inhibiting glucose transporter function. Furthermore van Can et al. [28] reported the implications for postprandial trehalose ingestion reduced glycemic responses in impaired glucose-tolerant subjects. Therefore a consuming trehalose with daily meals may contribute to the maintenance of good health through the prevention of hyperglycemia. Also trehalose may possibly contribute with useful saccharide in prediabetic and type 2 diabetic patients. These possibilities must be resolved in the future.

In conclusion, we have shown that it is hard for trehalose to give rise to hyperglycemia and hypersecretions of insulin and GIP following ingestion. These results indicate that trehalose may be a useful saccharide for good health because of properties that do not stimulate rapid increases in blood glucose and excessive secretion of insulin and GIP promoting fat accumulation.

## Abbreviations

GIP: Gastric inhibitory polypeptide
GLP-1: Glucagon-like peptide-1
DPP-4: Dipeptidyl peptidase-4
Tmax: Time to maximum concentrations
Cmax: Maximum concentrations
IAUC: Incremental area under the curve
BMI: Body mass index
